# Characterization of a novel ovine model of hypertensive heart failure with preserved ejection fraction

**DOI:** 10.1152/ajpheart.00548.2024

**Published:** 2024-11-15

**Authors:** Joshua W.-H. Chang, Siyi Chen, Charlotte Hamilton, Julia Shanks, Mridula Pachen, Audrys Pauza, Bindu George, Rohit Ramchandra

**Affiliations:** Manaaki Manawa – The Centre for Heart Research, Department of Physiology, University of Auckland, Auckland, New Zealand

**Keywords:** diastolic, exercise, hemodynamic, HFpEF, hypertension

## Abstract

The lack of animal models that accurately represent heart failure with preserved ejection fraction (HFpEF) has been a major barrier to the mechanistic understanding and development of effective therapies for this prevalent and debilitating syndrome characterized by multisystem impairments. Herein, we describe the development and characterization of a novel large animal model of HFpEF in older, female sheep with chronic 2-kidney, 1-clip hypertension. At 6-wk post unilateral renal artery clipping, hypertensive HFpEF sheep had higher mean arterial pressure compared with similarly aged ewes without unilateral renal artery clipping (mean arterial pressure = 112.7 ± 15.9 vs. 76.0 ± 10.1 mmHg, *P* < 0.0001). The hypertensive HFpEF sheep were characterized by *1*) echocardiographic evidence of diastolic dysfunction (lateral e′ = 0.11 ± 0.02 vs. 0.14 ± 0.04 m/s, *P* = 0.011; lateral E/e′ = 4.25 ± 0.77 vs. 3.63 ± 0.54, *P* = 0.028) and concentric left ventricular hypertrophy without overt systolic impairment, *2*) elevated directly measured left ventricular end-diastolic pressure (13 ± 5 vs. 0.5 ± 1 mmHg, *P* = 2.1 × 10^−6^), and *3*) normal directly measured cardiac output. Crucially, these hypertensive HFpEF sheep had impaired exercise capacity as demonstrated by their *1*) attenuated cardiac output (*P* = 0.001), *2*) augmented pulmonary capillary wedge pressure (*P* = 0.026), and *3*) attenuated hindlimb blood flow (*P* = 3.4 × 10^−4^) responses, during graded treadmill exercise testing. In addition, exercise renal blood flow responses were also altered. Collectively, our data indicates that this novel ovine model of HFpEF may be a useful translational research tool because it exhibits similar and clinically relevant impairments as that of patients with HFpEF.

**NEW & NOTEWORTHY** We show that older, female sheep with chronic 2-kidney, 1-clip hypertension have similar cardiac and noncardiac exercise hemodynamic abnormalities as patients with HFpEF. This clinically relevant, translatable, and novel large animal model of HFpEF may be useful for elucidating mechanisms and developing treatments for this increasingly common syndrome with few clinically impactful therapies.

## INTRODUCTION

Heart failure with preserved ejection fraction (HFpEF) is a clinical syndrome hallmarked by severe exercise intolerance with symptoms of breathlessness and fatigue on exertion during routine activities of daily living (1). HFpEF currently accounts for approximately half of all heart failure cases with this proportion increasing at around 1% per year to become the predominant form of heart failure (2). Symptoms of HFpEF occur despite a “normal” left ventricular ejection fraction (EF; ≥50%) and are the result of increased left ventricular filling pressure due to diastolic dysfunction (3). Importantly, exercise intolerance in HFpEF is now recognized to be a manifestation of impairments in not just the heart but also in other organs including the skeletal muscle and peripheral vasculature (3, 4). Furthermore, these impairments are frequently absent at rest and only become apparent during exertion when they are rapidly and markedly exacerbated (3).

Until recently, HFpEF has had no known treatments which have demonstrated a consistent or convincing prognostic benefit. This is because HFpEF is a heterogeneous syndrome with a complex and incompletely understood pathophysiology affecting multiple organ systems. This has resulted in a lack of animal models which accurately recapitulate HFpEF, creating a major barrier to our mechanistic understanding of the syndrome and the generation of effective therapies (4, 5).

Although multiple reviews on animal models of HFpEF are available (6, 7), the following summarizes why currently available models have limited clinical translatability: *1*) patients with HFpEF tend to be older, female, and highly comorbid, whereas animals used for HFpEF studies tend to be young and male with only one or two comorbidities, *2*) the majority of animal models are in rodents whose hearts are not comparable to that of humans in terms of size, morphology, and function, *3*) most animal models of HFpEF do not fully meet the clinical criteria for a HFpEF diagnosis and could therefore be considered extended models of left ventricular diastolic dysfunction rather than HFpEF, *4*) many animal models of HFpEF are known to have a later decrement in EF, whereas such a change rarely occurs in patients with HFpEF, and *5*) animal studies are frequently performed under the confounding effects of anesthesia which may alter function and/or lack functional or stress-based testing which is particularly relevant given that HFpEF is characterized by often normal function at rest but impaired function upon exertion, leading to exercise intolerance. The main aim of this study was therefore to develop and characterize an animal model of HFpEF that attempts to address these limitations.

Herein, we describe the generation and characterization of a novel large animal model of HFpEF in older, female sheep with chronic hypertension. Using a variety of methods, we show the presence of diastolic dysfunction and cardiac hypertrophy without overt systolic dysfunction. Crucially, through graded exercise testing, we demonstrate that the hemodynamic determinants which contribute to exercise capacity are impaired similarly to patients with HFpEF and that such impairments involve both cardiac and noncardiac (peripheral) structures. Finally, we report on the molecular signature of this novel model of HFpEF by exploring changes in the expression of select genes using qRT-PCR.

## MATERIALS AND METHODS

### Ethical Approval

**Figure 1.**
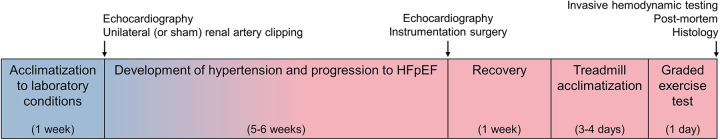
Timeline of the study protocol. HFpEF, heart failure with preserved ejection fraction.

Experiments were performed on aged (>5 yr old), female Romney sheep sourced from the University of Auckland Ngapouri Research Farm (49–73 kg). Sheep were housed in individual pens and metabolic cages and were acclimatized to laboratory conditions and human interaction before experimentation. Sheep were fed specially formulated pelleted feed (2–3 kg/day), water ad libitum, and supplemental hay and chaff as needed. All protocols were submitted to and approved by the University of Auckland Animal Ethics Committee. A timeline of the study protocol is shown in Fig. 1.

### Anesthesia

All surgical procedures were performed under anesthesia. After an overnight fast, sheep were given midazolam (0.25 mg/kg SC; Mylan) as premedication before the induction of anesthesia. Anesthesia was then induced with midazolam (0.15 mg/kg iv) and propofol (5 mg/kg iv; Fresenius Kabi, Austria) and following intubation, was maintained with an isoflurane-air-oxygen mixture (1.5–2.5%; Lunan Better Pharmaceutical, P.R. China) while under mechanical ventilation. Throughout all surgical procedures, the depth of anesthesia was regularly monitored by assessing the pedal and eyelash reflexes, and if required, the concentration of isoflurane was altered.

For all surgical procedures, sheep were also given a prophylactic antibiotic (oxytetracycline long-acting 20 mg/kg im; Kela, Belgium) and meloxicam (1 mg/kg iv; Boehringer Ingelheim, Germany), buprenorphine (0.01 mg/kg SC; Ceva, Australia), and fentanyl (50 μg/h transdermal patch; Sandoz, Germany) for analgesia. These medications were given before the first incision. Postoperative analgesia was managed by continuation of the fentanyl transdermal patch for 72 h and meloxicam which was given daily for a further 6 days following surgery.

### Ovine Model of Hypertensive Heart Failure with Preserved Ejection Fraction (Unilateral Renal Artery Clipping Surgery)

Chronic hypertension was surgically induced using the 2-kidney, 1-clip model in aged ewes. As described previously (8), a right flank skin incision was made followed by division of the subcutaneous fat. The abdominal muscles were then divided and underlying fat retracted to expose the right renal artery. A custom-made stainless steel clip was placed around this artery and tightened using a screwdriver so that blood flow to the right kidney was partially obstructed. The muscle layers were then separately closed before closure of the flank wound. Sheep were left to develop hypertension for 5–6 wk before undergoing echocardiography and instrumentation surgery in preparation for further experiments. Control (non-HFpEF) sheep were those that had their right renal artery isolated but without implantation of a renal artery clip (sham renal artery clipping).

Note that sheep were randomly allocated to either the unilateral renal artery clip or the sham renal artery clip group.

### Echocardiography

Echocardiography was performed immediately before unilateral (or sham) renal artery clipping and at 5–6 wk post unilateral (or sham) renal artery clipping immediately before instrumentation surgery. This was done under anesthesia using the Vivid S70N ultrasound system (GE HealthCare). Electrocardiograms were available, and scans were performed with reference to guidelines by the American Society of Echocardiography (9) and Vloumidi and Fthenakis (10), and guidance from an experienced clinical cardiac sonographer. Sheep were placed in left lateral recumbency and scanned from the right hemithorax. Two-dimensional and M-mode techniques were used to obtain the following parameters in both the parasternal short- and parasternal long-axis views: interventricular septum thickness at end-diastole (IVSd; cm), left ventricular internal diameter at end-diastole (LVIDd; cm), left ventricular posterior wall thickness at end-diastole (LVPWd; cm), and left ventricular internal diameter at end-systole (LVIDs; cm). Ejection fraction (EF; %) using the Teichholz method and relative wall thickness (RWT) were calculated by the built-in software of the ultrasound system. In the apical four-chamber view, pulsed-wave Doppler, with a maximal corrected angle of 40°, was used to record the mitral inflow signal for measurement of peak E-wave velocity (E; m/s) and peak A-wave velocity (A; m/s). Color Doppler was used to guide the placement of the pulsed-wave Doppler sample volume. In the same apical four-chamber view, pulsed-wave tissue Doppler imaging, with a maximal corrected angle of 40°, was used to record septal and lateral mitral annular velocities, from which early diastolic velocities (e′) were obtained. Mitral E/A, septal E/e′, lateral E/e′, and average E/e′ were calculated by the built-in software of the ultrasound system. All measurements were performed offline.

### Instrumentation Surgery

After at least 5 wk post unilateral renal artery clipping, all animals underwent instrumentation of the neck along with either instrumentation of the thorax (*n* = 15, non-HFpEF; *n* = 11, hypertensive HFpEF) or instrumentation of the abdomen (*n* = 11, non-HFpEF; *n* = 6, hypertensive HFpEF). Animals were given 7 days to recover from instrumentation surgery before undergoing graded exercise testing.

### Instrumentation of the Neck

Instrumentation of the neck was performed so that chronic vascular access and recording of blood pressure (BP) could be established. As described previously (8), a skin incision was made on the right side of the neck, and the underlying tissue was bluntly dissected to expose the right common carotid artery and right jugular vein. A cannula and a solid-state pressure catheter (SPR-524; Millar) were then separately inserted into the right common carotid artery and tunneled subcutaneously to the nape, whereas an 8-French size, 11 cm sheath introducer (504-608X; Cordis) was inserted into the right jugular vein before closure of the neck wound.

Chronic implantation of the sheath introducer into the right jugular vein ensured that access was available for pulmonary capillary wedge pressure (PCWP) measurements. On the day of graded exercise testing, a 6-French size pulmonary artery catheter with two lumens (TD2602NXF; Merit Medical Systems) was connected to a fluid-filled pressure transducer placed at the level of the heart. The pulmonary artery catheter was then introduced via the sheath introducer into the right jugular vein and advanced until a pulmonary artery waveform was obtained. Correct positioning of the pulmonary artery catheter was determined when the pulmonary artery waveform changed into a PCWP waveform following inflation of the pulmonary artery catheter balloon and vice versa when the balloon was deflated.

### Instrumentation of the Thorax

Instrumentation of the thorax was undertaken to facilitate the chronic recording of cardiac output (CO) and coronary artery blood flow (CoBF). First, intercostal nerve blocks were performed as an additional analgesic measure by injecting bupivacaine with adrenaline 0.25%/1:400,000 (3 mL per injection; Aspen, France) into the second to sixth intercostal spaces. A dorsal to ventral skin incision was then made over the left fourth rib and the subcutaneous fat was divided by electrocautery. Next, the underlying superficial muscles were bluntly dissected before the division of the deeper muscles overlying the left fourth rib. The periosteum of the left fourth rib was then stripped, the rib removed, and part of the left lung retracted to visualize the heart and its great vessels. At this stage, an incision was made in the pericardium and perivascular ultrasonic flow probes were placed around the ascending aorta (28PAU; Transonic Systems) and the left main coronary artery or left circumflex artery (6PS; Transonic Systems) for subsequent measurement of CO and CoBF, respectively. The pericardium and deep muscle layers were then separately closed, followed by restoration of negative intrathoracic pressure, subcutaneous tunneling of the flow probes, and closure of the thoracic wound.

### Instrumentation of the Abdomen

Instrumentation of the abdomen was performed in a separate group of animals to facilitate chronic recording of blood flow to the hindlimb and nonclipped (left) kidney. In a similar fashion to the unilateral renal artery clipping surgery, the abdominal cavity was accessed from the left flank and the distal (infrarenal) abdominal aorta and left renal artery were exposed. This facilitated the placement of perivascular ultrasonic flow probes around *1*) the distal abdominal aorta for subsequent measurement of hindlimb blood flow (HLBF; 20PAU; Transonic Systems) and *2*) the left renal artery for subsequent measurement of renal blood flow (RBF; 6PS; Transonic Systems). Note that if any arterial branches were observed between the left renal artery and the iliac bifurcation of the abdominal aorta, then these were ligated. Flow probes were then tunneled subcutaneously and the abdomen closed as per the renal artery clipping surgery.

### Exercise Capacity

**Figure 2.**
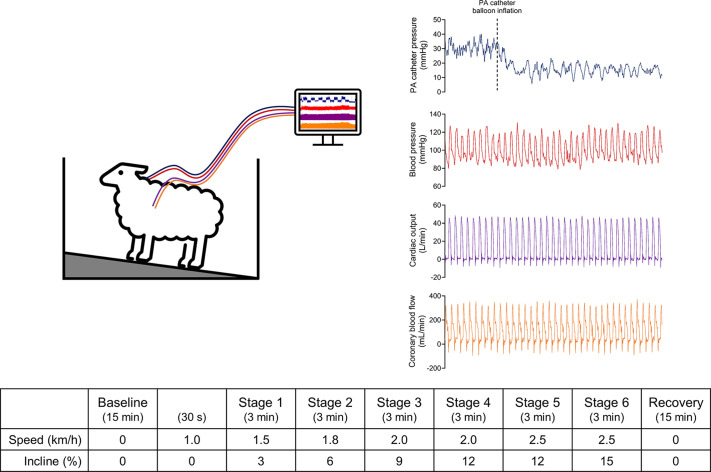
Graded treadmill exercise test protocol and representative 15-s beat-to-beat recording of blood pressure, cardiac output, coronary blood flow, and pulmonary capillary wedge pressure during exercise. PA, pulmonary artery.

Hemodynamic variables which contribute to exercise capacity were recorded while sheep underwent graded exercise testing on a treadmill (Fig. 2). The graded exercise test was performed after a minimum of 7 days of recovery from instrumentation surgery and following 3–4 days of acclimatization to the treadmill when sheep walked at low speeds and low inclines for up to 15 min/day. On the day of the exercise test, sheep stood at rest on the treadmill for 15 min for baseline (resting) measurements immediately before the commencement of the exercise test, which consisted of walking at 1 km/h at 0% incline for 30 s, 1.5 km/h at 3% incline for 3 min (stage 1), 1.8 km/h at 6% incline for 3 min (stage 2), 2.0 km/h at 9% incline for 3 min (stage 3), 2.0 km/h at 12% incline for 3 min (stage 4), 2.5 km/h at 12% incline for 3 min (stage 5), and 2.5 km/h at 15% incline for 3 min (stage 6). At the end of the exercise test, sheep stood on the treadmill for a 15-min recovery period.

In animals that underwent instrumentation of the thorax, the hemodynamic variables acquired during the graded exercise test were BP (mmHg), CO (L/min), CoBF (mL/min), and PCWP (mmHg) (Fig. 2). In animals which underwent instrumentation of the abdomen, the hemodynamic variables acquired were BP, HLBF (L/min), and RBF (mL/min). These variables were continuously recorded on a computer using a data acquisition unit (Micro1401-3; Cambridge Electronic Design, UK) and data acquisition software (Spike 2; Cambridge Electronic Design, UK). Mean arterial pressure (MAP; mmHg), heart rate (HR; beats/min), stroke volume (SV; mL), systemic vascular conductance (SVC; mL/min/mmHg), and coronary vascular conductance (CoVC; mL/min/mmHg), or MAP, HR, hindlimb vascular conductance (HLVC; mL/min/mmHg), and renal vascular conductance (RVC; mL/min/mmHg) were also computed using scripts on Spike2. Data from Spike2 were then exported into Microsoft Excel for subsequent analysis.

To determine the hemodynamic responses to graded exercise testing, the absolute change of each variable (except PCWP) in the last 60 s of each stage of exercise was compared with its respective 15-min baseline average. PCWP was analyzed by comparing *1*) the PCWP in the last 60 s of each stage of exercise with its 15-min baseline average and *2*) changes in PCWP (from baseline) in relation to changes in CO (from baseline) (Δ PCWP/Δ CO; mmHg/L/min) during the last 60 s of each stage of exercise.

### Final Hemodynamic Measurement, Euthanasia, and Postmortem Examination

At the end of the study, sheep were anesthetized and direct measurement of left ventricular end-diastolic pressure (LVEDP) was made by retrograde insertion of a solid-state pressure catheter from the left common carotid artery into the left ventricle. Correct positioning of the catheter was determined when the pressure waveform changed to reflect that of the left ventricle. The catheter was then left in place to obtain LVEDP by continuous recording of left ventricular pressure for a minimum of 10 min under stable anesthesia. Following this, sheep were euthanized with an overdose of sodium pentobarbitone (5 g iv; Provet, New Zealand). A postmortem examination was then performed whereby the heart and both kidneys were harvested and weighed, and the cardiac tissue was processed for histology and gene expression analysis.

### Histology

Histology was performed to assess cardiomyocyte size and myocardial collagen content. Once the sheep was euthanized, the heart was harvested and tissue from the lateral wall of the mid-left ventricle was taken. This tissue was fixed in a 1% paraformaldehyde solution at 4°C for 1 h. Cryoprotection was then carried out by placing the tissue at 4°C in a 10% sucrose solution for 1 h, 20% sucrose solution overnight, and 30% sucrose solution for at least 24 h until the tissue sunk. The tissue was then frozen at −80°C before subsequent processing.

### Cardiomyocyte Size and Myocardial Collagen Content

Fixed frozen samples of left ventricle were sliced on a cryostat to 10 µm thickness. Slices were then mounted on microscope slides before treatment in phosphate-buffered saline with Triton X-100 (1%; Thermo Fisher Scientific), then Image-iT FX Signal Enhancer (Thermo Fisher Scientific) for 1 h, and then wheat germ agglutinin (1:200; Thermo Fisher Scientific) in the dark for 2 h at room temperature. Final prepared sections were coverslipped with VECTASHIELD antifade mounting medium (Vector Laboratories) and were stored at 4°C for fluorescent imaging. Stained sections were imaged on a Zeiss LSM 710 confocal microscope. A total of 30 different myocytes, from 5 to 7 sections of the left ventricle of each animal, were randomly selected. Quantitative measurements of length, width, and area of each myocyte were performed using the noncommercial image processing software Fiji (11).

To measure collagen deposition in the left ventricle, 10 µm slices of the left ventricle were stained with Masson’s trichrome and then imaged and analyzed as previously reported (12).

### Gene Expression Analysis

The molecular signature of the sheep was assessed via steady-state gene expression analysis. During organ harvesting at the end of the study, left ventricular tissue biopsies were taken and snap-frozen in liquid nitrogen before storage at −80°C until batch processing. Samples were homogenized using 2.8 mm ceramic beads (Cat. No. 19-628; Omni International, GA) in QIAzol Lysis Reagent (Cat. No. 79306; QIAGEN, Germany) with an Omni Bead Ruptor 12 homogenizer (Omni International). RNA was then isolated using the Zymo Direct-zol RNA MicroPrep Kit (Cat. No. R2062; Zymo Research) per manufacturer’s instructions and an on-column DNAse treatment was performed for all samples. RNA was resuspended in nuclease-free water (Cat. No. AM9937; Thermo Fisher Scientific) and concentration was determined using the Qubit RNA High Sensitivity Assay Kit (Cat. No. Q32852; Thermo Fisher Scientific). RNA was reverse-transcribed using the iScript gDNA Clear cDNA Synthesis Kit (Cat. No. 1725035; Bio-Rad). gDNA removal step was included as part of cDNA synthesis protocol for every sample. qRT-PCR was carried out in duplicates using the Luna Universal qPCR Master Mix (Cat. No. M3003; New England Biolabs) on a QuantStudio 12 K Flex Real-Time PCR System (Thermo Fisher Scientific). *RPLP1* and *GAPDH* were used as housekeeping/loading controls which were included on the same plate as target genes. Melt curve was performed at the end of each run to confirm a single amplicon in each well. No template controls were included for every assay. For relative quantification of gene expression, the 2−ΔΔCT method was used (13). Primers used are summarized in Table 1.

**Table 1.** qRT-PCR primers used in the study

Target	Forward 5′-3′	Reverse 5′-3′
*MYH6*	ACGCTCTTCTCCTCGTATGC	ATGATGCAGCGCACAAAGTG
*TNNT2*	TGAGATGGTCAGACGGGCTA	CAGGTCCACAGCACAGAGAG
*KCNH2*	GGACACCATCATCCGCAAGT	AAGCCGTCGTTGCAGTAGAT
*PLN*	TTCACAACAGCCAAGGCTACC	AGCAGAGCGAGTGAGGTACT
*NDUFA5*	TGCTGAAGAAGACCACTGGC	GCTGCATTTTTAGGGATGTGTCC
*MECR*	GCCAAGGTCGTCGAACTGA	GGAAGGAGGCCGTAGTTTCC
*COQ5*	GGGCAAAGGTGAAAGGGTCTA	ACCGGAATGCAATGTCACCT
*MPC1*	GGGACTACCTCATGAGCACTTT	AAATGTCATCCGCCCACTGA
*SLC27A3*	GCAAAAGAGGGCTTTGACCC	ATCCCTAACAGCAGTTCCCC
*PPARG*	GATCTCCAGCGACATCGACC	CTCGCCTTTGCTTTGGTCAG
*LPL*	AGCCCCGGCTTTGATATTGG	TGCCAAGTTTCAGCCAGACT
*PLIN2*	ACTGGCTGGTAGGTCCCTTT	GGGTGTTTTCTACTCCGCCT
*SLC2A1*	TGTTGCCGGTTTCTCCAACT	AGAACAGAACCAGGAGCACG
*OAS1*	GCTTCTGACGGTCTATGCCT	CCTGGGTTTTGCAAGTTGCTT
*RPLP1*	CCCTCATTCTGCACGACGAT	AAGGCCTTTGCAAACAAACCT
*GAPDH*	TGGCCTCCAAGGAGTAAGGT	CAGGGCCTTGAGGATGGAAA

### Statistical Analysis

Minimum group sizes were determined based on preliminary data estimates of effect size for changes in all variables. All results are reported as mean (SD), and all variables were tested for normality. Differences in baseline measurements and exercise Δ PCWP/Δ CO slope between non-HFpEF and hypertensive HFpEF sheep were analyzed using the unpaired *t* test (two-tailed). Differences in the hemodynamic responses to graded exercise testing between non-HFpEF and hypertensive HFpEF sheep were analyzed using a two-way ANOVA where the independent factors were animal, stage of exercise, and group (hypertensive HFpEF/non-HFpEF); the effects of interaction term were examined. We also conducted a one-way ANOVA to assess the effects of exercise testing on the hemodynamic variables in each group. Hypothesis testing of gene expression data was performed using pairwise Wilcoxon–Mann–Whitney *U* tests on ΔCT values, while multiple comparison correction was performed using the Benjamini–Hochberg procedure (SPSS software; IBM). Precise *P* values are presented, and results were considered statistically significant if *P* < 0.05.

## RESULTS

### Baseline (Resting) Measurements

A summary of hemodynamic variables measured in conscious animals under resting conditions is presented in Table 2.

**Table 2.** Baseline measurements in non-HFpEF and hypertensive HFpEF sheep

	Non-HFpEF	*n*	Hypertensive HFpEF	*n*	*P* Value
Hemodynamic variable
Mean arterial pressure, mmHg	76.0 (10.1)	25	112.7 (15.9)	16	3.5 × 10^−11^
Heart rate, beats/min	98.7 (4.6)	26	96.8 (19.0)	17	0.71
Cardiac output, L/min	9.49 (1.71)	14	8.19 (1.38)	11	0.052
Stroke volume, mL	93.7 (19.6)	13	85.6 (18.9)	11	0.32
Systemic vascular conductance, mL/min/mmHg	126 (26.7)	13	74.9 (15.9)	10	2.7 × 10^−5^
Coronary blood flow, mL/min	93.2 (34.2)	13	98.8 (35.3)	10	0.71
Coronary vascular conductance, mL/min/mmHg	1.24 (0.51)	12	0.85 (0.26)	9	0.052
Hindlimb blood flow, L/min	1.61 (0.40)	11	1.74 (0.58)	6	0.57
Hindlimb vascular conductance, mL/min/mmHg	22.3 (8.3)	11	15.8 (6.6)	6	0.12
Renal blood flow, mL/min	734 (263)	10	766 (168)	6	0.80
Renal vascular conductance, mL/min/mmHg	10.5 (4.9)	10	6.8 (1.6)	6	0.10
Pulmonary capillary wedge pressure, mmHg	9.9 (2.0)	9	10.9 (2.9)	6	0.46
Left ventricular end-diastolic pressure, mmHg	0.5 (1)	8	13 (5)	6	2.1 × 10^−6^
Echocardiography
Ejection fraction, %	63 (8)	13	62 (8)	14	0.67
Interventricular septum thickness at end-diastole, cm	0.9 (0.2)	13	1.1 (0.2)	14	0.0039
Left ventricular internal diameter at end-diastole, cm	4.8 (0.4)	13	4.6 (0.4)	14	0.22
Left ventricular posterior wall thickness at end-diastole, cm	1.0 (0.2)	13	1.2 (0.2)	14	0.0035
Left ventricular internal diameter at end-systole, cm	3.1 (0.4)	13	3.0 (0.4)	14	0.51
Relative wall thickness	0.41 (0.08)	13	0.53 (0.10)	14	0.0035
Septal e′, m/s	0.09 (0.02)	12	0.09 (0.02)	14	0.50
Lateral e′, m/s	0.14 (0.04)	13	0.11 (0.02)	14	0.011
Septal E/e′	5.51 (1.27)	12	5.50 (1.44)	12	0.99
Lateral E/e′	3.63 (0.54)	13	4.25 (0.77)	12	0.028
E/A	1.46 (0.26)	13	1.16 (0.27)	11	0.012
Mass
Heart, g	423.8 (52.9)	7	491.6 (97.5)	10	0.12
Clipped (right) kidney, g	109.9 (15.3)	7	77.5 (15.0)	11	0.0004
Non-clipped (left) kidney, g	108.0 (14.5)	7	137.2 (20.2)	11	0.0045
Histology
Cardiomyocyte area, μm^2^	1654 (191)	9	2186 (601)	6	0.026
Cardiomyocyte width, μm	18.1 (1.9)	9	22.2 (4.8)	6	0.040
Cardiomyocyte length, μm	103.9 (8.4)	9	118.0 (22.9)	6	0.11
Myocardial collagen content, %	2.3 (0.78)	6	4.8 (2.1)	6	0.021

Values are mean (SD). All hemodynamic variables (except left ventricular end-diastolic pressure) were measured in conscious animals under resting conditions. Echocardiography parameters (and left ventricular end-diastolic pressure) were obtained under anesthesia. Mass of the heart and kidneys were measured at postmortem. The unpaired *t* test was used for comparisons between non-HFpEF and hypertensive HFpEF sheep. HFpEF, heart failure with preserved ejection fraction.

Sheep that underwent unilateral renal artery clipping (hypertensive HFpEF group) had higher MAP and lower SVC than sheep in the control (non-HFpEF) group. There was otherwise no difference in any of the other variables measured at rest (e.g., CO) between the two groups.

Postmortem weights showed that while the right and left kidneys of the non-HFpEF animals were comparable in weight (*P* = 0.82), unilateral renal artery clipping was associated with a reduction in the weight of the clipped (right) kidney and a compensatory enlargement of the nonclipped (left) kidney (Table 2).

### Echocardiography

**Figure 3.**
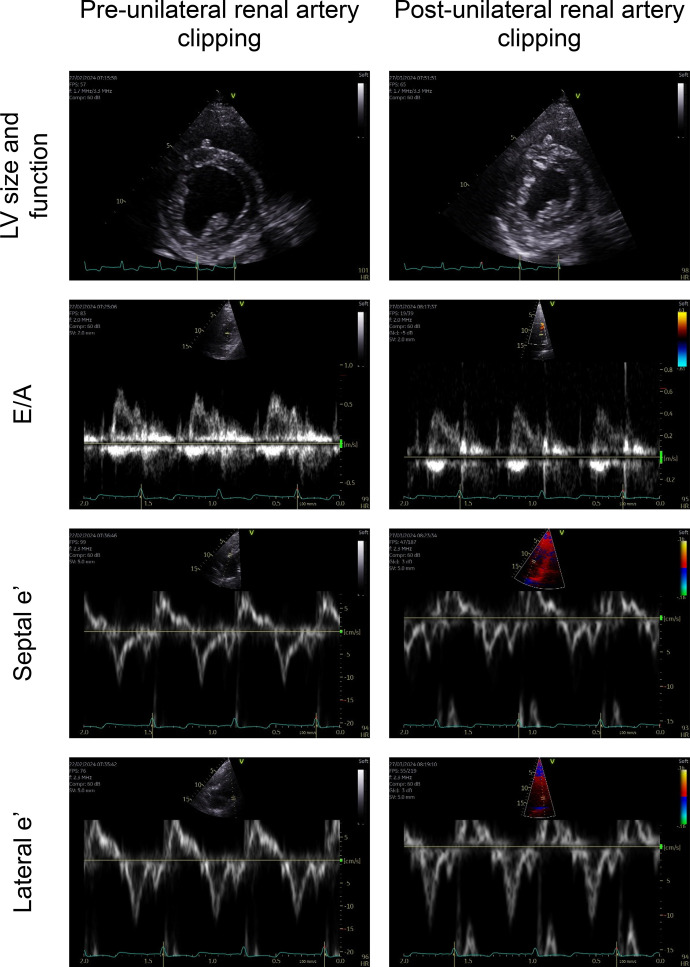
Examples of echocardiography images obtained under anesthesia from one animal pre- and postunilateral renal artery clipping. E/A, ratio of early to late diastolic transmitral flow velocity; e′, early diastolic mitral annular velocity; LV, left ventricle.

Echocardiography parameters obtained under anesthesia are presented in Table 2, and examples of echocardiography images obtained from one animal pre- and postunilateral renal artery clipping are shown in Fig. 3.

There was no difference in EF between non-HFpEF and hypertensive HFpEF sheep. Compared with non-HFpEF sheep, hypertensive HFpEF sheep had echocardiographic evidence of concentric left ventricular hypertrophy based on their greater IVSd, LVPWd and RWT, and unchanged LVIDd. Hypertensive HFpEF sheep also had echocardiographic evidence of diastolic dysfunction based on their lower lateral e′ and higher lateral E/e′. This was supported by direct measurements made at the end of the study showing an elevated LVEDP in this group.

### Histology

**Figure 4.**
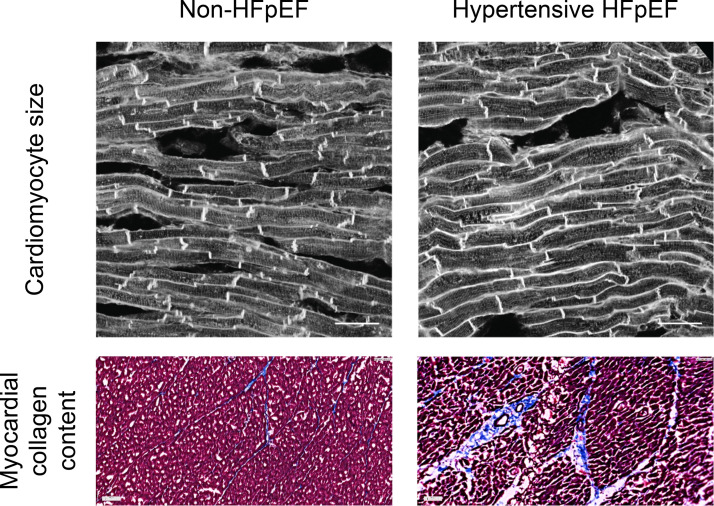
Examples of histological sections obtained for the analysis of cardiomyocyte size and myocardial collagen content in non-HFpEF and hypertensive HFpEF sheep (scale bars = 50 μm). HFpEF, heart failure with preserved ejection fraction.

Examples of histological sections obtained from non-HFpEF and hypertensive HFpEF sheep are shown in Fig. 4. Consistent with the echocardiographic findings, histological analysis of cardiac tissue from the hypertensive HFpEF animals revealed the presence of cardiomyocyte hypertrophy and myocardial fibrosis (Table 2). In the hypertensive HFpEF animals, cardiomyocytes exhibited an increase in cell area that was a result of an increase in cell width rather than cell length. In addition, myocardial collagen content was higher in the hypertensive HFpEF animals.

### Exercise Capacity

Hemodynamic variables which contribute to exercise capacity were continuously recorded on a beat-to-beat basis during graded exercise testing (Fig. 2).

### Blood Pressure, Heart Rate, and Systemic Vascular Conductance Responses to Exercise

**Figure 5.**
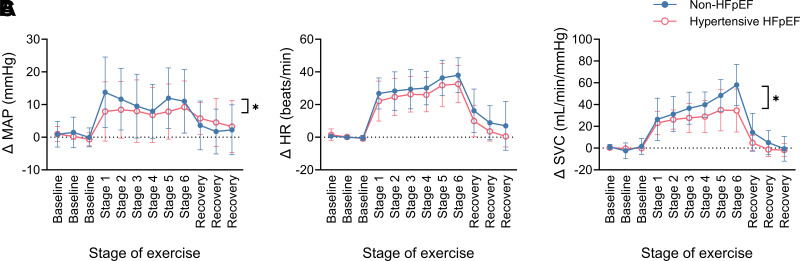
Absolute change in mean arterial pressure (MAP; non-HFpEF *n* = 25, HFpEF *n* = 16; *P = 0.025, two-way ANOVA interaction effect; *A*), heart rate (HR; non-HFpEF *n* = 26, HFpEF *n* = 17; *B*), and systemic vascular conductance (SVC; non-HFpEF *n* = 13, HFpEF *n* = 10; *P = 0.002, two-way ANOVA interaction effect; *C*) during the graded treadmill exercise test in non-HFpEF (blue, closed circles) and hypertensive HFpEF sheep (pink, open circles). Data are mean (SD). HFpEF, heart failure with preserved ejection fraction.

At the onset of exercise, there was an immediate increase in MAP which remained elevated throughout the graded exercise test (Fig. 5*A*). In contrast, HR increased progressively with increasing exercise intensity and there was no difference in the HR response between hypertensive HFpEF and non-HFpEF animals (Fig. 5*B*). Similarly, SVC progressively increased with increasing exercise intensity. However, this response was attenuated in the hypertensive HFpEF animals (Fig. 5*C*).

### Cardiac Output and Coronary Blood Flow Responses to Exercise

**Figure 6.**
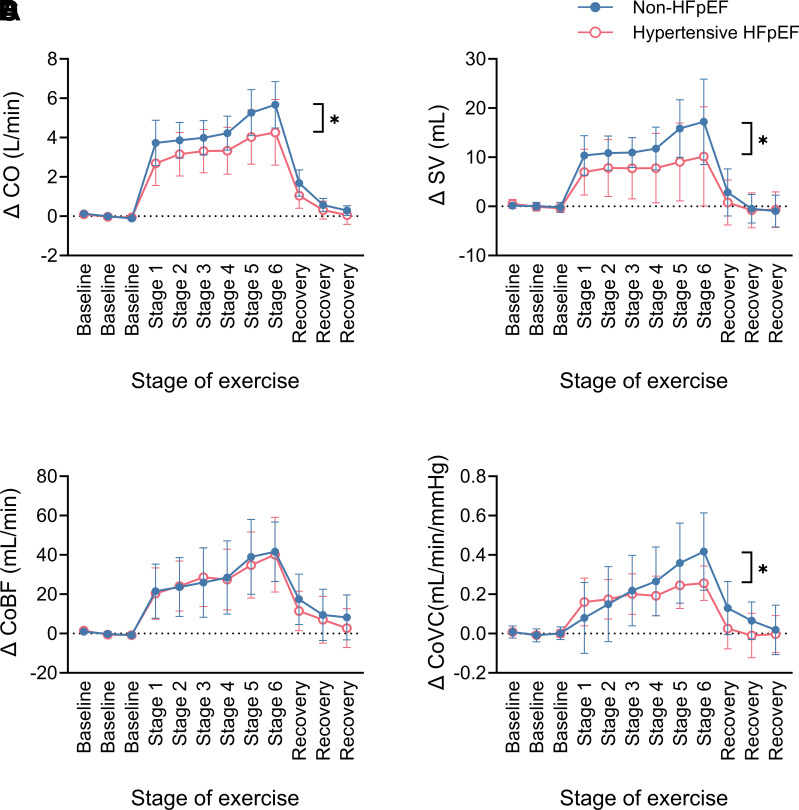
Absolute change in cardiac output (CO; non-HFpEF *n* = 14, HFpEF *n* = 11; *P = 0.001, two-way ANOVA interaction effect; *A*), stroke volume (SV; non-HFpEF *n* = 13, HFpEF *n* = 11; *P = 0.002, two-way ANOVA interaction effect; *B*), coronary blood flow (CoBF; non-HFpEF *n* = 13, HFpEF *n* = 10; *C*), and coronary vascular conductance (CoVC; non-HFpEF *n* = 12, HFpEF *n* = 9; *P = 0.001, two-way ANOVA interaction effect; *D*) during the graded treadmill exercise test in non-HFpEF (blue, closed circles) and hypertensive HFpEF sheep (pink, open circles). Data are mean (SD). HFpEF, heart failure with preserved ejection fraction.

Increasing exercise intensity was associated with progressive increases in CO. However, the exercise CO response was attenuated in the hypertensive HFpEF animals (Fig. 6*A*) due to a diminished SV (Fig. 6*B*), but not HR, response. Accordingly, although there was no difference in the progressive increase in coronary blood flow (CoBF) between the hypertensive HFpEF and non-HFpEF animals during graded exercise testing (Fig. 6*C*), increases in exercise CoVC were attenuated in the hypertensive HFpEF animals (Fig. 6*D*).

### Pulmonary Capillary Wedge Pressure Response to Exercise

**Figure 7.**
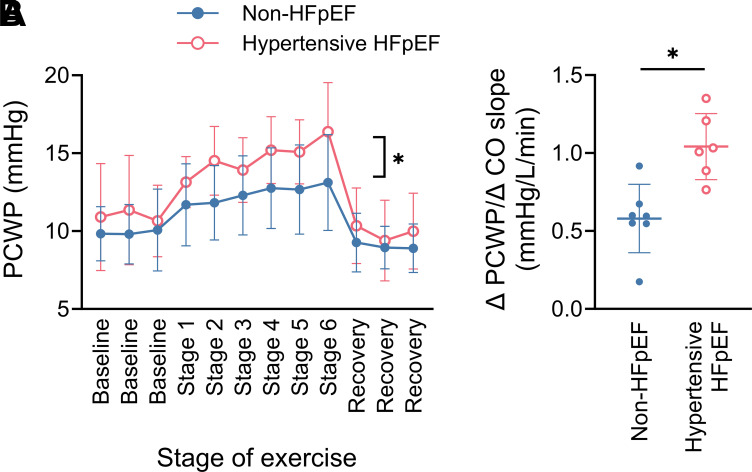
Pulmonary capillary wedge pressure (PCWP; non-HFpEF *n* = 9, HFpEF *n* = 6; *P = 0.026, two-way ANOVA interaction effect; *A*) and the change in PCWP for any given change in cardiac output (Δ PCWP/Δ CO slope; non-HFpEF *n* = 7, HFpEF *n* = 6; *P = 0.0027, unpaired *t* test; *B*) during the graded treadmill exercise test in non-HFpEF (blue, closed circles) and hypertensive HFpEF sheep (pink, open circles). Data are mean (SD). HFpEF, heart failure with preserved ejection fraction.

PCWP progressively increased during the graded exercise test. At each stage of exercise, hypertensive HFpEF animals had higher PCWP compared with non-HFpEF animals (Fig. 7*A*). Moreover, during exercise, hypertensive HFpEF animals had greater increases in PCWP for any given increase in CO (Fig. 7*B*).

### Hindlimb and Renal Blood Flow Responses to Exercise

**Figure 8.**
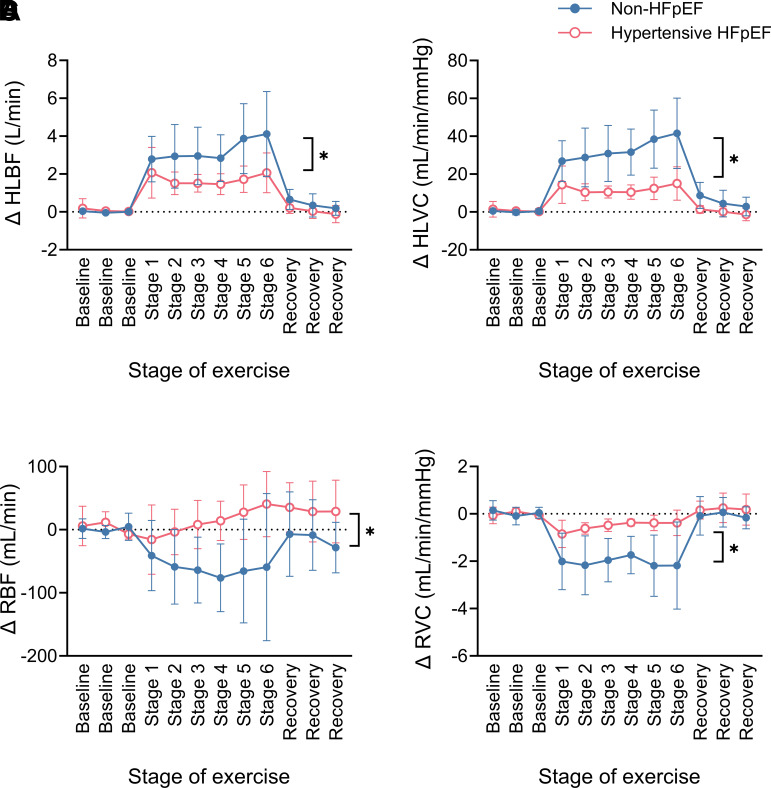
Absolute change in hindlimb blood flow (HLBF; non-HFpEF *n* = 11, HFpEF *n* = 6; *P = 3.4 × 10^−4^, two-way ANOVA interaction effect; *A*), hindlimb vascular conductance (HLVC; non-HFpEF *n* = 11, HFpEF *n* = 6; *P = 6.8 × 10^−8^, two-way ANOVA interaction effect; *B*), renal blood flow (RBF; non-HFpEF *n* = 10, HFpEF *n* = 6; *P = 0.005, two-way ANOVA interaction effect; *C*), and renal vascular conductance (RVC; non-HFpEF *n* = 10, HFpEF *n* = 6; *P = 3.0 × 10^−9^, two-way ANOVA interaction effect; *D*) during the graded treadmill exercise test in non-HFpEF (blue, closed circles) and hypertensive HFpEF sheep (pink, open circles). Data are mean (SD). HFpEF, heart failure with preserved ejection fraction.

Exercise was associated with increases in HLBF and HLVC; however, these responses were attenuated in the hypertensive HFpEF animals (Fig. 8, *A* and *B*).

Similarly, hypertensive HFpEF and non-HFpEF animals had differing exercise RBF and RVC responses (Fig. 8, *C* and *D*). In the non-HFpEF animals, exercise was associated with a decrease in RBF and RVC. In contrast, although the onset of exercise in the hypertensive HFpEF animals was associated with a smaller decrease in RBF and RVC, this was transient as further increases in exercise intensity were associated with a gradual increase in RBF and RVC. Notably, hypertensive HFpEF animals had higher RBF during the two highest levels of exercise intensity when compared with baseline (resting) and an attenuated decrease in RVC throughout the graded exercise test.

### Gene Expression (Molecular Signature)

**Figure 9.**
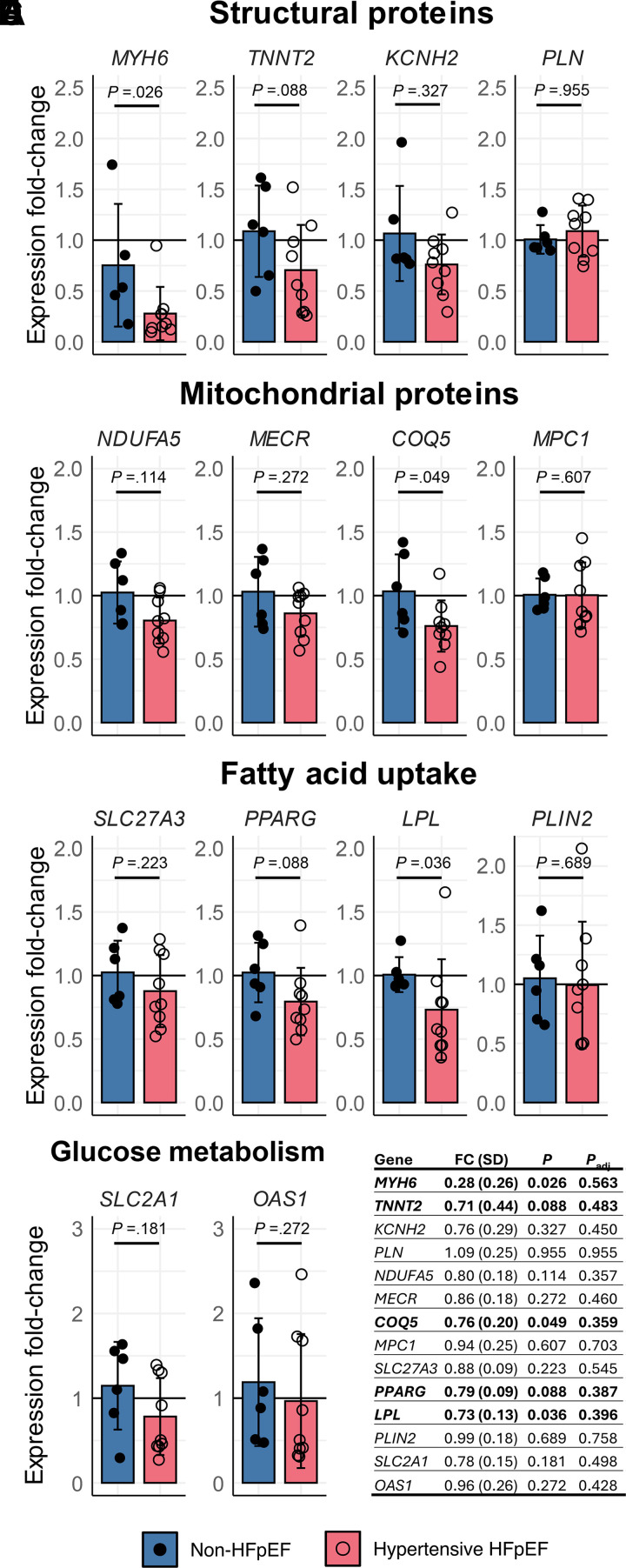
Fold-change (FC) in gene expression between non-HFpEF (blue, closed circles; *n* = 6) and hypertensive HFpEF (pink, open circles; *n* = 9) sheep. The genes were selected based on their roles in structural proteins (*A*), mitochondrial proteins (*B*), fatty acid uptake (*C*), and glucose metabolism(*D*). *E*: *P* values associated with these comparisons using the Wilcoxon–Mann–Whitney *U* test as well as adjusted *P* values using the Benjamini–Hochberg procedure. Data are mean (SD). *COQ5*, coenzyme Q5, methyltransferase; HFpEF, heart failure with preserved ejection fraction; *KCNH2*, potassium voltage-gated channel subfamily H member 2; *LPL*, lipoprotein lipase; *MECR,* mitochondrial trans-2-enoyl-CoA reductase; *MPC1*, mitochondrial pyruvate carrier 1; *MYH6*, myosin heavy chain 6; *NDUFA5*, NADH:ubiquinone oxidoreductase subunit A5; *OAS1*, 2'-5'-oligoadenylate synthetase 1; *PLN*, phospholamban; *PLIN2*, perilipin 2; *PPARG*, peroxisome proliferator activated receptor gamma; *SLC27A3*, solute carrier family 27 member 3; *SLC2A1*, solute carrier family 2 member 1; *TNNT2,* troponin T2, cardiac type.

The expression of 14 target genes that have previously been shown to be altered in clinical samples as well as animal models of HFpEF (14–17) was tested using qRT-PCR. The gene targets were selected to represent distinct aspects of cardiac function in HFpEF as detailed in Fig. 9. Overall, HFpEF was associated with the downregulation of three out of the four gene groups tested. Specifically, our model was associated with a reduction in levels of *MYH6* (myosin heavy chain 6) involved in cardiac structure, a reduction in *COQ5* (coenzyme Q5, methyltransferase) involved in mitochondrial function, and a decrease in *LPL* (lipoprotein lipase) which is involved in breaking down fat in the form of triglycerides. No changes in genes involved in glucose metabolism were observed. However, when multiple comparison correction was applied to account for the number of genes examined, none of the expression changes detected passed the *P* < 0.05 significance threshold.

## DISCUSSION

The primary objective of this study was to develop and characterize a novel animal model of HFpEF in an attempt to address the shortcomings of current existing models of HFpEF. In doing so, this would produce a clinically relevant and translatable animal model of HFpEF that permits future mechanistic and interventional study.

The main finding of this study is that both cardiac and noncardiac (peripheral) abnormalities which characterize human HFpEF can be reproduced in a large animal model possessing multiple HFpEF risk factors. Specifically, chronic hypertension which was surgically induced using the 2-kidney, 1-clip model in aged ewes caused concentric left ventricular hypertrophy (increased IVSd, LVPWd, and RWT on echocardiography and increased cardiomyocyte size seen in histology) and diastolic dysfunction (decreased lateral e′ and increased lateral E/e′ on echocardiography and increased LVEDP measured directly) without overt systolic impairment (normal and unchanged left ventricular EF and resting CO). Moreover, abnormal exercise hemodynamics (exaggerated PCWP and impaired CO, vasodilatory and skeletal muscle blood flow reserve capacities), which are known contributors to exercise intolerance in HFpEF patients, were also present in this model of HFpEF as demonstrated via graded exercise testing.

Importantly, our study findings and the clinical relevance of the present animal model of HFpEF are highlighted when diagnostic HFpEF algorithms are applied. For example, using the HFA-PEFF score (18), our model would score at least three points (2 points for a reduction in lateral e′ plus 1 point for echocardiographic evidence of concentric hypertrophy). With this score, the HFA-PEFF diagnostic algorithm advocates for a diastolic stress test or invasive hemodynamic measurements, where we clearly demonstrate that hypertensive HFpEF sheep have substantially elevated LVEDP and abnormal stress (exercise) responses.

### Hemodynamic Determinants of Exercise Capacity in HFpEF

Exercise intolerance is a cardinal feature of HFpEF. Accordingly, a novel aspect and strength of the present study was the ability to assess exercise capacity in a large animal model by characterizing the hemodynamic reserve capacity of multiple organ systems during graded exercise testing. Researchers have previously developed large animal models of HFpEF in pigs utilizing varied and multiple insults (19–21). However, an advantage of our model is the relative ease with which sheep can be handled in the laboratory setting. In this context, we are, to our knowledge, the first to perform direct, continuous, and real-time hemodynamic measurements during exercise in a large animal model of HFpEF.

In patients with HFpEF, the cardiac output (CO) response (reserve) to exercise is impaired and this has been directly correlated with the exercise intolerance that patients with HFpEF experience (22, 23). A substantial contributor to this blunted CO response is an increase in left ventricular filling pressure, which is a universal and key defining feature of HFpEF that may only be evident on exertion during the early stages of the syndrome (3). Consistent with this, the hypertensive HFpEF animals in this study had normal CO and pulmonary capillary wedge pressure (PCWP; surrogate for left ventricular filling pressure) at rest, but attenuated CO (Fig. 6*A*) and augmented PCWP responses and a steeper rise in PCWP for any given increase in CO during exercise (Fig. 7, *B* and *C*), suggesting that this is a model of early HFpEF.

Impairments in the other determinants of CO also contribute to its diminished reserve in HFpEF, although their relative contributions to exercise intolerance may differ between patients (24). In addition to diastolic dysfunction (which causes increased left ventricular filling pressure), patients with HFpEF may exhibit chronotropic incompetence (slower rise in HR, lower peak HR, and impaired HR recovery) and impaired cardiac contractility during exercise (23, 25–29). Comparably, although the hypertensive HFpEF animals in the present study did not exhibit an abnormal HR response to exercise, their SV response was attenuated.

Importantly, the contribution of noncardiac (peripheral) factors to exercise intolerance in HFpEF has become increasingly recognized and may play a relatively greater role than cardiac factors in limiting exercise capacity in patients with HFpEF (30). For example, diminished systemic vasodilator reserve (23, 25) and impaired blood flow to exercising skeletal muscle (31–34) have been implicated in the pathophysiology of exercise intolerance in HFpEF patients. Similarly, the hypertensive HFpEF animals in the current study had blunted systemic vasodilation during exercise. This included the diminished capacity of the hindlimb vasculature to vasodilate which was associated with an attenuated increase in hindlimb blood flow (HLBF) during exercise. With respect to the latter, impaired blood flow to exercising skeletal muscle in patients with HFpEF is frequently unrelated to changes in central hemodynamic variables such as CO (31). Our data indicates that, at least in this model of HFpEF, the impaired HLBF reserve capacity was partly due to insufficient redistribution of blood flow from the kidneys to the hindlimbs as a result of inadequate renal vasoconstriction during exercise. This is in stark contrast to heart failure with reduced ejection fraction (HFrEF) where there is augmented renal vasoconstriction during exercise (35, 36), suggestive of excessive sympathetic nerve activity to the kidneys. In comparison, whether increases in sympathetic nerve activity to the kidney are attenuated and/or its transduction to a vascular response is impaired in the present model of HFpEF remains to be directly tested.

Finally, the CoVC response to exercise was attenuated in the hypertensive HFpEF animals of the present study. In patients with HFpEF, reduced myocardial (coronary) blood flow reserve has emerged as a potential pathophysiological factor for HFpEF (37, 38). Whilst the hypertensive HFpEF animals in the current study did not demonstrate an impaired coronary blood flow (CoBF) response to exercise per se, the fact that CoBF increased similarly to the non-HFpEF animals during exercise despite a similar HR but lower CO response suggests that there may be inefficiency in cardiac function in the hypertensive HFpEF animals. However, this needs to be directly tested.

### Molecular Signature of HFpEF

Previous studies have proposed that different phenotypes of HFpEF may present with distinct molecular signatures which reflect the primary pathology (e.g., chronic hypertension, renal impairment, type 2 diabetes mellitus, and obesity) underlying diastolic dysfunction and an HFpEF diagnosis (14, 39). To explore the molecular signature of the present model, we tested a set of genes that have previously been shown to be altered in myocardial biopsies obtained from patients with HFpEF and animal models of HFpEF, and which represent hallmark molecular signatures of HFpEF pathology. Although only a limited number of genes were tested, this was sufficient to demonstrate that our HFpEF model exhibits transcriptional remodeling of cardiac tissue.

Interestingly, we observed downregulation of the gene encoding for *COQ5* (coenzyme Q5, methyltransferase) which is involved in the biosynthesis of CoQ10 and ATP production in the mitochondria. We also observed a reduction in the gene encoding for *LPL* (lipoprotein lipase) which has a crucial role in lipid metabolism and transport (40). The downregulation of *MYH6* which encodes for myosin heavy chain 6 was expected given the cardiac hypertrophy. It is also known that the heart switches from glucose to fatty acid utilization for myocardial energy production in a preclinical model of HFpEF (41). However, given high fat was not a factor that was deliberately considered in the generation of the present model, the downregulation of *COQ5* and *LPL* seems surprising. Therefore, our data raises the question of whether changes in these pathways are a consequence of diastolic dysfunction and HFpEF rather than resulting in HFpEF. Indeed, a recent study which showed no difference in right ventricular biopsies from patients with HFpEF with prominent obesity and diabetes versus predominantly hypertension and left ventricular hypertrophy would be in agreement with this (14). Nonetheless, we do note the exploratory nature of the gene expression analysis performed herein given that correcting for multiple comparisons meant there were no longer any statistically significant changes.

### Limitations

First, the present animal model should be viewed as one representing a hypertensive HFpEF phenotype rather than one that seeks to replicate HFpEF associated with the metabolic syndrome. However, we demonstrate that chronic hypertension, combined with other common risk factors for HFpEF such as the female sex and older age in sheep, is sufficient to reproduce many of the key exertional hemodynamic abnormalities observed in patients with HFpEF, including that observed in hypertensive but non-obese HFpEF patients (42, 43). Concordant with this, the relatively modest exaggeration in exercise PCWP in the present animal model reflects that of hypertensive, non-obese HFpEF patients who have smaller increases in exercise PCWP than that of patients with HFpEF who are both hypertensive and obese (43). Comparatively, chronic hypertension induced in adult female canines via similar means as the present study does not tend to alter the CO and HLBF responses to exercise (44–47). The reasons for such a discrepancy between the present study and the canine studies are unclear but may relate to *1*) species differences and *2*) the possibility that the hypertensive HFpEF animals in the present study had additional factors that predisposed them to a HFpEF phenotype, such as older age, potential obesity (49–73 kg in the present study vs. 20–25 kg in the canine studies) and the longer duration of hypertension (>42 days in the present study vs. >30 days in the canine studies).

Second, the severity of HFpEF represented in the present study is likely that of early HFpEF. In early HFpEF, hemodynamic abnormalities are usually absent at rest and only unmasked through exercise stress testing, as they are in the current study. Although our echocardiography data only showed changes in lateral, but not septal, e′ and E/e′, this may relate to the influence of the right ventricle on septal e′ (48) and/or the use of isoflurane anesthesia during echocardiography, which has previously been shown to prevent the detection of diastolic dysfunction in rodent HFpEF as compared with ketamine/xylazine anesthesia (49). Nonetheless, invasively measured LVEDP at the end of our study protocol was elevated in the hypertensive HFpEF animals, thus directly proving the presence of diastolic dysfunction. Left ventricular EF was also unchanged at the end of our study, although we acknowledge it is unknown if the present model would eventually progress to HFrEF.

Finally, most preclinical HFpEF studies either do not use female animals or do not consider sex differences (6). Thus, while the present animal model of HFpEF is female-specific, this, in fact, reflects the female preponderance in human HFpEF (2) and highlights the clinical relevance of the animal model presented herein.

## DATA AVAILABILITY

Data will be made available upon reasonable request.

## GRANTS

The study was supported by Health Research Council of New Zealand, Clinical Research Training Fellowship, Grant/Award Number: 22/071 (to J.W.-H.C.); Health Research Council of New Zealand, Sir Charles Hercus Fellowship, Grant/Award Number: 23/119 (to J.S.); Health Research Council of New Zealand, Project Grant, Grant/Award Number: 20/158 (to R.R.) and 24/215 (to J.S./R.R.); and Heart Foundation of New Zealand, Project Grant, Grant/Award Number: 1904 (to R.R.).

## DISCLOSURES

No conflicts of interest, financial or otherwise, are declared by the authors.

## AUTHOR CONTRIBUTIONS

J.W.-H.C. and R.R. conceived and designed research; J.W.-H.C., S.C., C.H., J.S., M.P., A.P., and B.G. performed experiments; J.W.-H.C., S.C., C.H., J.S., M.P., and A.P. analyzed data; J.W.-H.C., S.C., C.H., M.P., and R.R. interpreted results of experiments; J.W.-H.C., A.P., and R.R. prepared figures; J.W.-H.C. drafted manuscript; J.W.-H.C., J.S., M.P., A.P., B.G., and R.R. edited and revised manuscript; J.W.-H.C., J.S., M.P., A.P., B.G., and R.R. approved final version of manuscript.
